# Cyclin-dependent kinase 2 protects podocytes from apoptosis

**DOI:** 10.1038/srep21664

**Published:** 2016-02-15

**Authors:** Pauliina Saurus, Sara Kuusela, Vincent Dumont, Eero Lehtonen, Christopher L. Fogarty, Mariann I. Lassenius, Carol Forsblom, Markku Lehto, Moin A. Saleem, Per-Henrik Groop, Sanna Lehtonen

**Affiliations:** 1 Department of Pathology, University of Helsinki, 00290 Helsinki, Finland; 2 Laboratory Animal Centre, University of Helsinki, 00290 Helsinki Finland; 3 Folkhälsan Institute of Genetics, Folkhälsan Research Center, University of Helsinki, 00014 Helsinki, Finland; 4 Division of Nephrology, Helsinki University Hospital, 00290 Helsinki, Finland; 5 Diabetes & Obesity Research Program, Research Program´s Unit, 00014 University of Helsinki, Finland; 6 University of Bristol, Bristol Royal Hospital for Children, BS2 8BJ Bristol, United Kingdom; 7 Baker IDI Heart & Diabetes Institute, VIC 3004 Melbourne, Australia

## Abstract

Loss of podocytes is an early feature of diabetic nephropathy (DN) and predicts its progression. We found that treatment of podocytes with sera from normoalbuminuric type 1 diabetes patients with high lipopolysaccharide (LPS) activity, known to predict progression of DN, downregulated CDK2 (cyclin-dependent kinase 2). LPS-treatment of mice also reduced CDK2 expression. LPS-induced downregulation of CDK2 was prevented *in vitro* and *in vivo* by inhibiting the Toll-like receptor (TLR) pathway using immunomodulatory agent GIT27. We also observed that CDK2 is downregulated in the glomeruli of obese Zucker rats before the onset of proteinuria. Knockdown of CDK2, or inhibiting its activity with roscovitine in podocytes increased apoptosis. CDK2 knockdown also reduced expression of PDK1, an activator of the cell survival kinase Akt, and reduced Akt phosphorylation. This suggests that CDK2 regulates the activity of the cell survival pathway via PDK1. Furthermore, PDK1 knockdown reduced the expression of CDK2 suggesting a regulatory loop between CDK2 and PDK1. Collectively, our data show that CDK2 protects podocytes from apoptosis and that reduced expression of CDK2 associates with the development of DN. Preventing downregulation of CDK2 by blocking the TLR pathway with GIT27 may provide a means to prevent podocyte apoptosis and progression of DN.

Glomerular diseases, including diabetic nephropathy (DN), are the leading causes of end-stage renal disease^1^. The pathophysiological mechanisms leading to the development of DN are poorly characterized, but recent studies indicate that podocyte injury is involved^2^. Podocytes are highly specialized cells that are required for normal glomerular integrity. One of the key features of progressive glomerulosclerosis is podocyte loss due to apoptosis or detachment. Podocyte loss has been reported in Pima Indians with type 2 diabetes (T2D) and also in patients with type 1 diabetes (T1D)^3^ or T2D with or without DN^4^,^5^,^6^. However, our knowledge of the pathophysiological mechanisms and proteins involved in the regulation of podocyte apoptosis remains limited.

Several factors have been shown to induce podocyte injury, including high glucose, reactive oxygen species (ROS), transforming growth factor beta (TGF-β), angiotensin II, and lipopolysaccharides (LPS)/endotoxins^7^,^8^,^9^,^10^,^11^,^12^, that are lipid-soluble membrane components of the gram-negative bacteria. Interestingly, our previous study shows that normoalbuminuric T1D patients who progress to microalbuminuria have higher serum LPS activity than normoalbuminuric nonprogressors^13^. This indicates that high LPS activity in serum of patients with T1D is associated with the development of microalbuminuria^13^ and suggests that high LPS activity may induce podocyte injury in patients with T1D.

CDK2, a 42-kDa S-phase cyclin-dependent kinase, is activated by cyclin E in late G1 and by cyclin A in S phase where it controls G1/S passage^14^. CDK2 was believed to play an essential role in cell cycle progression, but CDK2 knockout mice are viable and have minimal phenotypic abnormalities^15^. It has been postulated that at least *in vivo*, other CDKs and cyclins may compensate for the missing CDKs^16^,^17^,^18^, however, this is still poorly understood. Nevertheless, it was shown that primary mouse embryonic fibroblasts isolated from CDK2 knockout mice show lower proliferation rates compared to wild type mouse embryonic fibroblasts^15^,^19^, suggesting that other CDKs are not able to efficiently compensate for the lack for CDK2. Previous studies addressing the role of CDK2 in the kidney have concentrated on proliferative podocyte injury models. In experimental glomerulonephritis, where podocytes re-enter the cell cycle and start to proliferate, inhibition of CDK2 has been shown to improve renal function by inhibiting proliferation of podocytes^20^. Inhibition of CDK2 has also been shown to decrease mesangial and glomerular endothelial cell proliferation in passive Heymann nephritis without aggravating podocyte damage^21^. Since mature podocytes are terminally differentiated and therefore have a limited capacity to proliferate, the role of CDK2 in healthy, nonproliferating podocytes and nonproliferative glomerular diseases, such as DN, remains unclear.

Since phosphoinositide 3-kinase (PI3K)-dependent Akt signaling pathway has a pivotal role in regulating cell survival^22^,^23^,^24^, the molecules modulating its activity may play key roles in regulating podocyte apoptosis. Akt is known to phosphorylate CDK2 *in vitro* in 293T cells, and transient activation of the pathway is necessary for cell cycle progression^25^,^26^. Interestingly, CDK2 promotes activation of Akt by phosphorylating residues Ser477 and Thr479 under cell cycle progression in HeLa cells^27^. However, constitutively activated Akt/CDK2 pathway promotes cell death^26^. Other studies point out that CDK2 may be either pro- or antiapoptotic apparently depending on the cellular status and the apoptotic stimulus^28^,^29^. We hypothesized that CDK2 could protect podocytes from apoptosis by promoting the activation of the Akt signaling pathway, and that podocyte injury induced by high LPS activity could downregulate CDK2 and subsequently enhance podocyte apoptosis.

## Results

### CDK2 is expressed in podocytes

**Figure 1:**
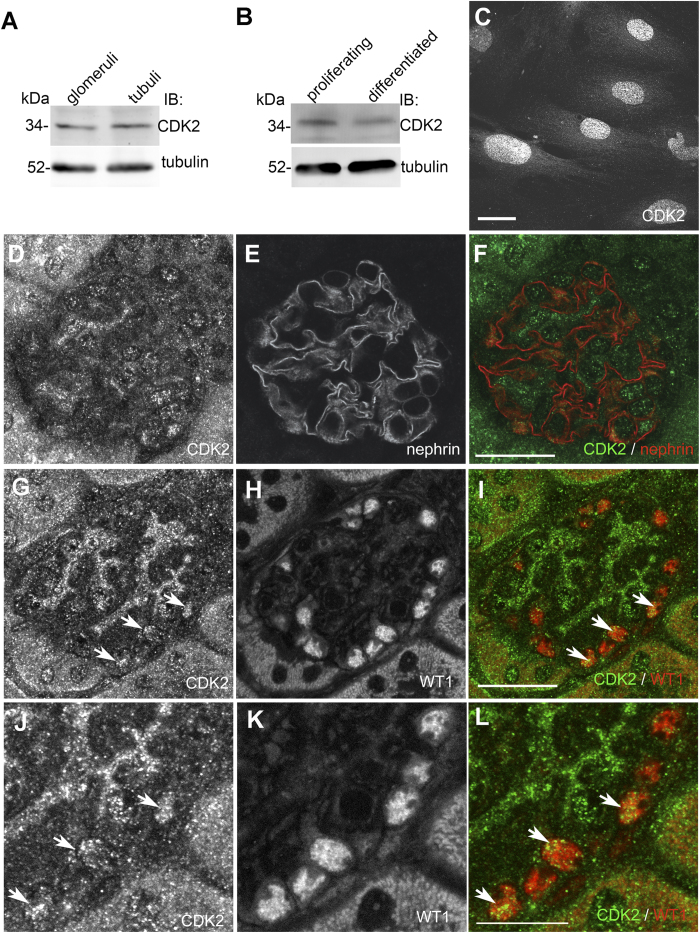
CDK2 is expressed in podocytes. (**A**) Immunoblot of rat glomerular and tubular fractions shows that CDK2 is expressed in glomeruli and tubuli. Tubulin is included as a loading control. (**B**) Immunoblot of cultured human podocytes shows that CDK2 is expressed in both proliferating and differentiated podocytes. Tubulin is included as a loading control. (**C**) Immunofluorescence microscopy indicates that CDK2 localizes mainly in the nuclei in differentiated human podocytes. (**D–F**) Perfused mouse kidney sections stained with CDK2 (**D**) and nephrin (**E**) antibodies shows that CDK2 is expressed in mouse glomerulus. (**G–L**) Perfused mouse kidney sections stained with CDK2 (**G**,**J**) and WT1 (**H**,**K**) antibodies shows that CDK2 concentrates in nuclei (arrows) in podocytes as visualized in merged image (**I**,**L**). Scale bars: (**C**) 12 μm, (**D–I**) 25 μm, (**J–L**) 12 μm.

Although CDK2 is widely expressed in human tissues, and Hiromura *et al.*^30^ showed CDK2 expression in both proliferating and differentiated mouse podocytes *in vitro*^30^, its localization and exact function in the glomerular podocytes remains unclear. We immunoblotted isolated rat glomerular and tubular fractions with an antibody against CDK2 and found that CDK2 is expressed in both glomeruli and tubules (Fig. 1A). Immunoblotting of lysates prepared from cultured human podocytes revealed that CDK2 is expressed in both proliferating and differentiated podocytes (Fig. 1B), and immunofluorescence microscopy indicated that CDK2 concentrates in the nuclei in differentiated podocytes (Fig. 1C). Double labeling of mouse kidney sections for CDK2 and nephrin showed that CDK2 is expressed in podocytes, and is also detected in other glomerular cells (Fig. 1D–F). Double labeling of mouse kidney sections for CDK2 and Wilms tumor 1 (WT1) confirmed that CDK2 is expressed in the nuclei of podocytes (Fig. 1G–L). Omission of the primary antibody showed no signal in the nuclei in glomeruli confirming the specificity of the staining (Supplementary Fig. S1).

### CDK2 expression is downregulated in the glomeruli of diabetic rats

**Figure 2:**
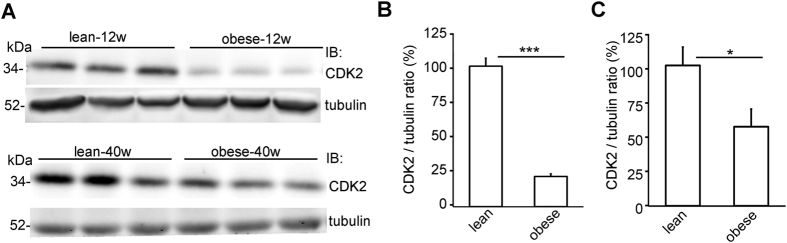
CDK2 is downregulated in the glomeruli of obese Zucker rats. (**A**) The expression of CDK2 is decreased in the glomeruli of 12 and 40 weeks old obese Zucker rats compared to lean controls. Tubulin is included as a loading control. (**B**) Quantification of CDK2 in the glomeruli of 6 individual lean and 6 individual obese 12 weeks old Zucker rats shows lower expression of CDK2 in the glomeruli of obese Zucker rats. (**C**) Quantification of CDK2 in the glomeruli of 6 individual lean and 6 individual obese 40 weeks old Zucker rats shows that the expression of CDK2 is lower in the glomeruli of obese Zucker rats. In (**A**), glomeruli were isolated, lysed and immunoblotted with anti-CDK2 IgGs. Data are presented as mean ± SEM (n = 6 per group). *p < 0.05, ***p < 0.001 vs. lean group.

Since the role of CDK2 in diabetes and DN has not been previously studied, we next investigated the expression level of CDK2 in the glomeruli of insulin resistant obese Zucker rats, a well-characterized model of T2D that shows similarities to early human DN^31^. At both 12 and 40 weeks of age, the obese rats had slightly higher blood glucose levels than the controls, but this did not reach statistical significance (Supplementary Table S1). At 12 weeks of age, the obese Zucker rats had higher urine albumin/creatine ratio than the lean controls, but the value was lower than in 40 weeks old lean rats (Supplementary Table S1), indicating that 12 weeks old rats did not have proteinuria. At 40 weeks, the obese rats had developed proteinuria (Supplementary Table S1). Quantitative Western blot analysis revealed that the expression level of CDK2 was decreased by 75% in the glomeruli of 12 weeks old (Fig. 2A,B) and by 45% in the glomeruli of 40 weeks old obese Zucker rats (Fig. 2A,C) when compared with lean controls.

### CDK2 is downregulated in podocyte injury models *in vitro*

**Figure 3:**
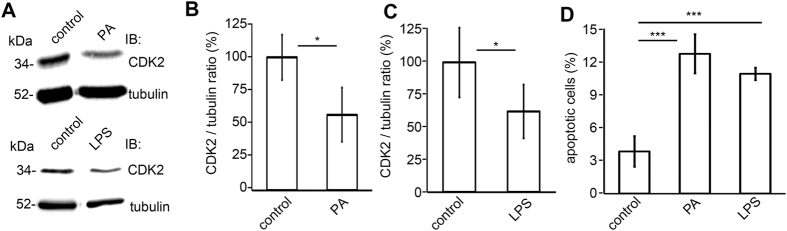
CDK2 is downregulated in podocyte injury models *in vitro*. (**A**) Representative immunoblot for CDK2 after PA- and LPS-treatments. Tubulin is included as a loading control. (**B**) Quantification of CDK2 after PA-treatment shows a decrease in CDK2 expression level in cultured human podocytes. (**C**) Quantification of CDK2 after LPS-treatment shows a decrease in CDK2 expression level in cultured human podocytes. (**D**) Flow cytometry of cultured human podocytes stained with annexin V and 7-AAD double labeling, where annexin V is used as an apoptosis and 7-AAD as a necrosis marker, confirms that PA- and LPS-treatments increase podocyte apoptosis. The experiments were performed three times with three replicates in each experiment. Data are presented as mean ± SD. *p < 0.05, ***p < 0.001 vs. control group.

Since CDK2 expression was decreased in the glomeruli of obese Zucker rats compared to controls, we next investigated whether CDK2 is downregulated in different podocyte injury models *in vitro*. Puromycin aminonucleoside (PA) and LPS are known inducers of podocyte injury and apoptosis both *in vitro* and *in vivo*^32^,^33^,^34^,^35^. PA is a toxin used to induce experimental minimal change nephrosis in rats^36^, and LPS is an endotoxin from gram-negative bacteria that induces sepsis and proteinuria in mice and rats^12^,^37^,^38^. PA-treatment of cultured human podocytes for 48 hours decreased the expression level of CDK2 by 45% (Fig. 3A,B). Treatment of podocytes with LPS for 48 hours also decreased the expression level of CDK2 by 35% (Fig. 3A,C). Fluorescence activated cell sorting (FACS) with annexin V and 7-Aminoactinomycin-D (7-AAD) double labelling, where annexin V is used as an apoptotic and 7-AAD as a necrotic marker, indicated that the level of apoptosis in control cells was 4%, whereas treatment with PA and LPS increased the level of apoptotic cells to 13% and 11%, respectively (Fig. 3D). The number of necrotic cells was not increased by LPS- or PA-treatment (2–3% in both).

### Inhibition of the TLR pathway restores the expression of CDK2 and reduces podocyte apoptosis induced by LPS

**Figure 4:**
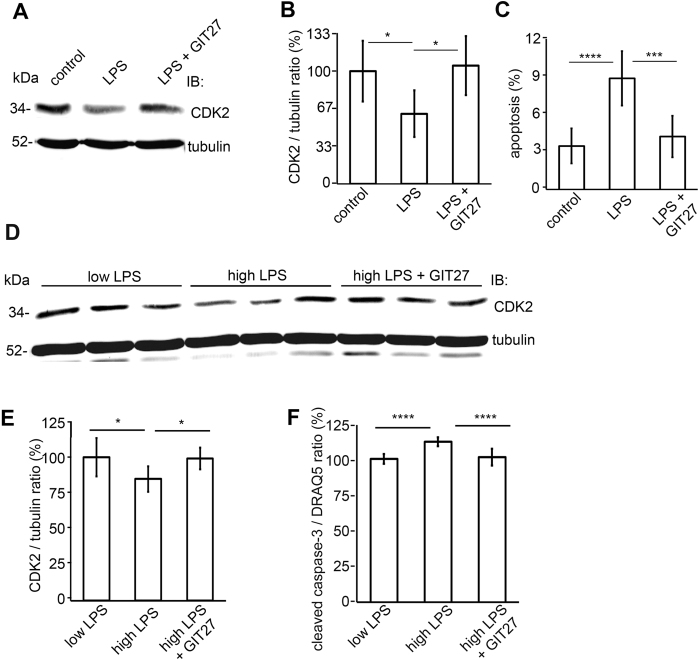
Inhibition of the TLR pathway prevents downregulation of CKD2 and induction of apoptosis in cultured human podocytes treated with LPS or with human sera with high LPS activity. (**A**) Representative immunoblot for CDK2 of LPS-treated podocytes with or without GIT27 treatment. Tubulin is included as a loading control. (**B**) Quantification of CDK2 shows that co-treatment of podocytes with LPS and GIT27 prevents downregulation of CDK2. The experiment was performed three times with three replicates in each experiment. Data are presented as mean ± SD. *p < 0.05 vs. LPS group. (**C**) Flow cytometry of podocytes stained for Annexin V confirms that co-treatment of podocytes with GIT27 and LPS prevents induction of apoptosis. The experiment was performed three times with three replicates in each experiment. Data are presented as mean ± SD. ***p < 0.001, ****p < 0.0001 vs. LPS group. (**D**) Representative immunoblot for CDK2 in cultured human podocytes treated with sera with low or high LPS activity, and with or without GIT27 co-treatment. Tubulin is included as a loading control. (**E**) Quantification of CDK2 from podocytes treated with sera with high or low LPS activity (n = 6 each), or treated with sera with high LPS activity in the presence of GIT27 (n = 6). The expression of CDK2 was lower after treatment with sera with high LPS activity than with low LPS activity. GIT27-treatment prevented downregulation of CDK2 induced by high LPS activity. The experiment was performed three times. Data are presented as mean ± SD. *p < 0.05, vs. high LPS group. (**F**) Quantification of In-Cell Western of cleaved caspase-3 in podocytes treated with sera with high or low LPS activity with or without GIT27 treatment shows that the expression of cleaved caspase-3 was higher after treatment with high-LPS sera compared to treatment with low-LPS sera. GIT27-treatment prevented the induction of apoptosis. DRAQ5^TM^ was used for normalization. Treatments were performed with sera from individual patients (n = 6 per group). The experiment was performed three times with 32 replicates in each group. Data are presented as mean ± SD, ****p < 0.0001 vs. high LPS group.

Podocytes recognize LPS by Toll-like receptor 4 (TLR4)^32^,^39^,^40^. In order to study whether the LPS-induced reduction in the expression of CDK2 is restored by TLR blockage, podocytes were treated with TLR pathway inhibitor 4,5-Dihydro-3-phenyl-5-isoxazoleacetic acid (GIT27) before addition of LPS to the media. Treatment of podocytes with LPS reduced the expression of CDK2 by 40% and increased apoptosis as determined by FACS analysis of Annexin V labeled cells (Fig. 4A–C). Co-treatment with GIT27 restored the expression of CDK2 and prevented podocyte apoptosis induced by LPS (Fig. 4A–C). Treatment with GIT27 alone had no effect to the expression level of CDK2 (Supplementary Figure S2).

To test the hypothesis that high serum LPS activity in T1D patients reduces the expression of CDK2, we supplemented the culture medium of human podocytes with sera from normoalbuminuric patients with high or low LPS activity. Patient data is described in Supplementary Table S2. Quantitative Western blotting revealed that the expression level of CDK2 was downregulated by 23% after 72h exposure to serum from patients with high LPS activity when compared to cells treated with serum from patients with low LPS activity (Fig. 4D,E). Sera with high LPS activity also increased apoptosis as indicated by increased level of cleaved caspase-3 (Fig. 4F). In order to confirm that CDK2 downregulation is mediated by endotoxins, cultured human podocytes were treated with GIT27 before adding the media with sera from normoalbuminuric patients with high serum LPS activity. Co-treatment with GIT27 restored the expression of CDK2 and reduced podocyte apoptosis (Fig. 4D–F).

### High glucose decreases CDK2 expression *in vitro*

We also tested whether high glucose treatment reduces CDK2 expression in cultured human podocytes, since high glucose has been shown to induce podocyte apoptosis^41^,^42^. The expression level of CDK2 was decreased slightly, by 13%, in podocytes treated with high glucose compared to podocytes cultured in normal glucose (Supplementary Fig. S3).

### Inhibition of the TLR pathway restores the expression of CDK2 in kidney and prevents podocyte foot process widening induced by LPS in mice

**Figure 5:**
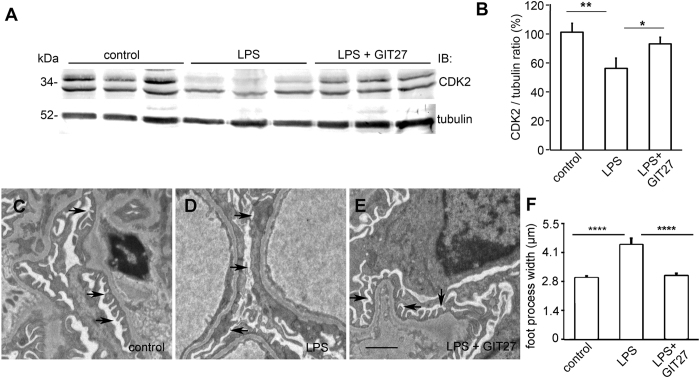
Inhibition of the TLR pathway prevents LPS-induced downregulation of CDK2 and podocyte foot process widening in BALB-C mouse kidneys. (**A**) Representative immunoblot for CDK2 in control, LPS-treated and LPS- and GIT27-treated mouse kidney cortical lysates. Tubulin is included as a loading control. (**B**) Quantification of CDK2 in mouse kidney cortical lysates shows that co-treatment of mice with LPS and GIT27 prevents LPS-induced downregulation of CDK2 (n = 6/treatment group). Data are presented as mean ± SEM. *p < 0.05, **p < 0.01 vs. LPS group. (**C**) Electron microscopy of control mouse kidney shows podocyte foot processes (arrows) regularly lining the glomerular basement membrane around capillary loops. (**D**) LPS-treatment induces podocyte foot process widening (arrows). (**E**) GIT27 co-treatment prevents LPS-induced podocyte foot process widening (arrows). Scale bar (**C–E**): 2 μm. (**F**) Quantification of podocyte foot process width confirms that LPS causes foot process widening which is prevented by GIT27 co-treatment. Foot process width was calculated from 4 animals per group, 3 glomeruli per animal and 3 capillary loops per glomeruli. Data are presented as mean ± SEM. ****p < 0.0001 vs. LPS group. In (**A**), mouse kidney cortices were lysed and immunoblotted with anti-CDK2 IgG.

To investigate the effect of LPS on CDK2 expression *in vivo* and whether lowered CDK2 expression can be rescued by inhibiting the TLR pathway as *in vitro*, we used LPS to induce podocyte injury in BALB-C mice as previously described^43^. Mice were treated with either GIT27 or its vehicle 24 h prior and 2 h after LPS challenge. Control mice received vehicle only. Albuminuria, CDK2 expression and podocyte foot process effacement were analyzed 24 h after LPS challenge. LPS increased urinary albumin excretion from 0.16 ± 0.15 mg/mmol to 21.27 ± 10.01 mg/mmol (p < 0.05). LPS also decreased the expression of CDK2 in mouse kidneys as observed by immunoblotting of kidney cortical lysates (Fig. 5A,B). The two bands observed by immunoblotting apparently represent differentially phosphorylated forms of CDK2 as previously described^44^. In addition, immunohistochemistry revealed reduction of CDK2 in the glomeruli of LPS-treated mice (Supplementary Fig. S4A,B). Co-treatment with GIT27 prevented downregulation of CDK2 in the kidney (Fig. 5A,B) and glomeruli (Supplementary Fig. S4A,C). GIT27 also decreased urinary albumin excretion from 21.27 ± 10.01 mg/mmol to 10.88 ± 1.85 mg/mmol, but this did not reach statistical significance. Electron microscopy analysis of control, LPS-treated and LPS- and GIT27-treated mouse kidneys revealed irregular widening of the podocyte foot processes in the LPS-treated mice (Fig. 5C,D,F). This was prevented by GIT27 co-treatment (Fig. 5D– F).

### Knockdown of CDK2 by shRNAs or inhibiting the activity of CDK2 by roscovitine increases apoptosis of cultured human podocytes

**Figure 6:**
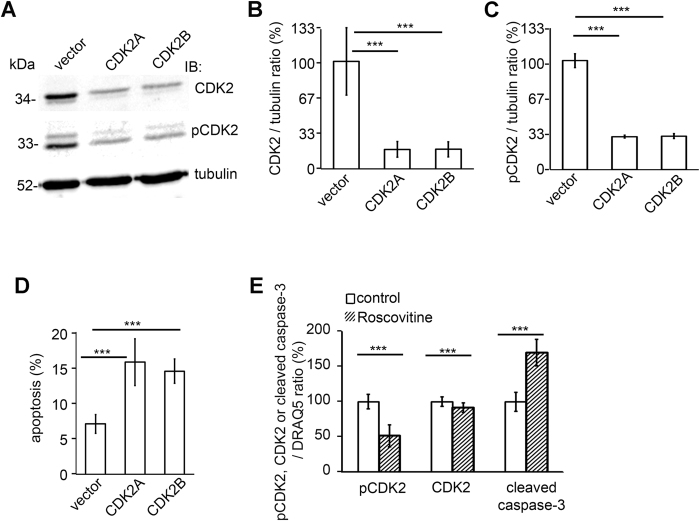
Knockdown of CDK2 by shRNAs increases apoptosis in cultured human podocytes. (**A**) Representative immunoblot for CDK2 and phospho-CDK2 (pCDK2, Thr160) in CDK2 knockdown cells. Podocytes were infected using two different shRNA constructs targeting CDK2 (CDK2A, CDK2B). Empty vector (vector) shRNA served as a control. Tubulin is included as a loading control. (**B**) CDK2 protein level is significantly decreased by both shRNAs compared to empty vector shRNA. (**C**) Quantification of phospho-CDK2 shows that the phosphorylation level of CDK2 is downregulated after knockdown of CDK2. The experiments (**A**,**B**) were performed three times with three replicates in each experiment. (**D**) Flow cytometry of podocytes stained with annexin V confirms that CDK2 knockdown increases podocyte apoptosis. The experiment was performed three times with three replicates in each experiment. Data are presented as mean ± SD. ***p < 0.001 vs. vector group. (**E**) Quantification of In-Cell Western of phospho-CDK2, CDK2 and cleaved caspase-3 in podocytes treated or not with 25 μM roscovitine shows that the expression of phosphorylated CDK2 and CDK2 are decreased and the expression of cleaved caspase-3 is higher after treatment with roscovitine compared to control. DRAQ5^TM^ was used for normalization. The experiment was performed three times, with 24 replicates per group. Data are presented as mean ± SD, ***p < 0.001 vs. control group.

Reduction of the expression level of CDK2 in podocytes after treatment with apoptosis-inducing agents PA and LPS suggests that CDK2 could be involved in the regulation of apoptosis in podocytes. To confirm this hypothesis, CDK2 was knocked down in cultured human podocytes using two different lentiviral small hairpin RNAs (shRNAs), CDK2A and CDK2B. Immunoblotting showed that both shRNAs lowered CDK2 protein level by 80% ± 5% and the level of active, phosphorylated CDK2 (Thr160) by 70% ± 0.8% 48 h after infection compared to podocytes infected with viruses carrying the empty vector shRNA (Fig. 6A,B,C). FACS-analysis with annexin V and 7-AAD confirmed that the level of apoptosis was increased from 5–7% in podocytes infected with the empty vector to 15–17% in podocytes infected with CDK2A or CDK2B shRNAs (Fig. 6D). The level of apoptosis in untreated control cells was similar to cells infected with the empty vector (4–8% in both), and the rate of necrotic cells was not increased by CDK2 knockdown (0.3% in both CDK2 shRNA-infected and empty vector-infected podocytes).

To further confirm the role of CDK2 in podocyte apoptosis, we inhibited the kinase activity of CDK2 by using roscovitine that is a well known inhibitor of CDK2 activity^14^,^21^. Inhibition of CDK2 activity using roscovitine in cultured human podocytes decreased the phosphorylation level of CDK2 (Thr160) by 49 ± 15% and the expression level of CDK2 by 10% ± 7%. Interestingly, roscovitine increased apoptosis of podocytes as visualized by 69% ± 18% increase in the expression level of cleaved caspase-3 (Fig. 6E). Collectively, either depletion of CDK2 or inhibition of its kinase activity enhances podocyte apoptosis.

### Knockdown of CDK2 downregulates cyclin E and inhibits the antiapoptotic and stimulates the proapoptotic pathways

**Figure 7:**
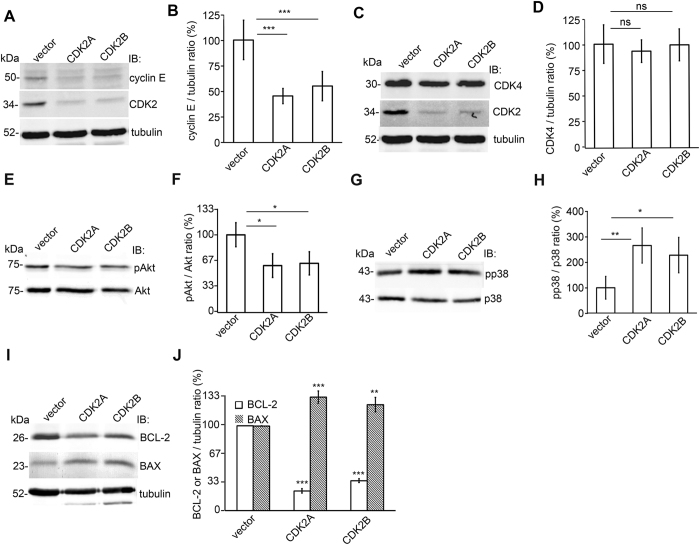
CDK2 knockdown inhibits cyclin E and the antiapoptotic pathways and stimulates the pro-apoptotic pathways in cultured human podocytes. (**A**) Immunoblot of cyclin E and CDK2 after knockdown of CDK2 with two different shRNA constructs (CDK2A and CDK2B) in cultured podocytes. Empty vector (vector) served as a control. Tubulin is included as a loading control. (**B**) Quantification of cyclin E shows that the expression level of cyclin E is downregulated after knockdown of CDK2. (**C**) Immunoblot of CDK4 after CDK2 knockdown in cultured podocytes. Tubulin is included as a loading control. (**D**) Quantification of CDK4 shows that the expression level of CDK4 does not change after CDK2 knockdown confirming the specificity of the knockdown. (**E**) Immunoblot assay of phosphorylated Akt (pAkt) in podocytes after CDK2 knockdown. Total Akt is included as a loading control. (**F**) Quantification of phosphorylated Akt shows that CDK2 knockdown decreases Akt activity in cultured podocytes. (**G**) Immunoblot of phosphorylated p38 (pp38) in podocytes after CDK2 knockdown. Total p38 is included as a loading control. (**H**) Quantification of phosphorylated p38 shows that CDK2 knockdown increases p38 activity in cultured podocytes. (**I**) Immunoblot of BCL-2 and BAX after knockdown of CDK2 in cultured podocytes. Tubulin is included as a loading control. (**J**) Quantification of BCL-2 and BAX shows that the expression level of BCL-2 is downregulated and BAX upregulated after knockdown of CDK2. Each experiment was performed three times with three replicates in each experiment. Data are presented as mean ± SD. ns: non significant, *p < 0.05, **p < 0.01, ***p < 0.001 vs. vector group.

In order to study the molecular pathways involved in apoptosis induced by low CDK2 expression, we examined the expression of cyclin E, an activator of CDK2^14^,^15^, cyclin-dependent kinase 4 (CDK4), another cyclin-dependent kinase, and apoptosis-associated kinases Akt and p38MAPK, after CDK2 knockdown. We found that CDK2 knockdown reduced the expression of cyclin E by 45–55% (Fig. 7A,B). CDK2 knockdown had no effect on CDK4 expression confirming the specificity of the knockdown (Fig. 7C,D). In addition, knockdown of CDK2 reduced the phosphorylation level of Akt on Ser473 by 35–40% (Fig. 7E,F), and increased the activation of the proapoptotic p38 MAPK pathway by inducing phosphorylation of p38 by 150–180% (Fig. 7G,H).

To investigate the involvement of proteins of the intrinsic apoptotic pathway in apoptotic processes involving CDK2, we studied the expression level of BCL-2 and BAX after CDK2 knockdown. Depletion of CDK2 decreased the level of BCL-2 by 80% and increased the level of BAX by 30–35% (Fig. 7I,J) indicating that the expression level of CDK2 influences the expression levels of the pro-survival protein BCL-2 and the pro-apoptotic protein BAX.

### Knockdown of CDK2 reduces PDK1 expression and PDK1 knockdown reduces CDK2 expression

**Figure 8:**
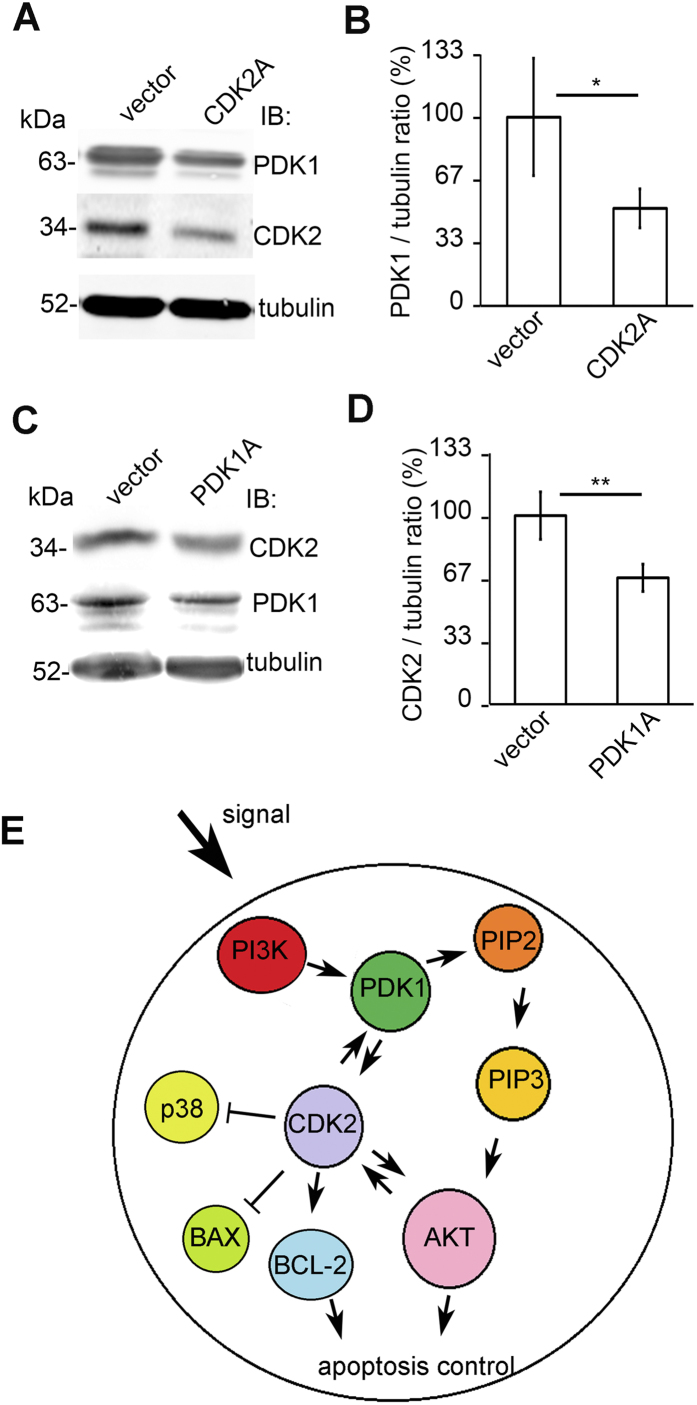
CDK2 knockdown reduces PDK1 expression and PDK1 knockdown reduces CDK2 expression in cultured human podocytes. (**A)** Representative immunoblot for PDK1 showing decreased PDK1 expression after CDK2 knockdown. Tubulin is included as a loading control. (**B**) Quantification of PDK1 shows that CDK2 knockdown decreases PDK1 expression in cultured podocytes. (**C**) Representative immunoblot for CDK2 after PDK1 knockdown in podocytes showing decreased CDK2 expression. Tubulin is included as a loading control. (**D**) Quantification of CDK2 shows that PDK1 knockdown decreases CDK2 expression in cultured podocytes. Each experiment was performed three times with three replicates in each experiment. Data are presented as mean ± SD. *p < 0.05, **p < 0.01 vs. vector group. (**E**) Schematic illustration linking CDK2 to the PI3K-dependent Akt signalling in podocyte apoptosis. PI3K stimulates PDK1, which activates Akt by phosphorylation of PIP2 to PIP3. PDK1 also induces CDK2 expression and CDK2 induces PDK1 expression. CDK2 reduces p38 phosphorylation and BAX expression and induces BCL-2 expression and Akt phosphorylation. In addition, Akt is known to activate CDK2. Induced BCL-2 expression and Akt phosphorylation protect podocytes from apoptosis. PIP2: phosphoinositol(4,5)biphosphate; PIP3: phosphoinositol(3,4,5)trisphosphate.

Since downregulation of CDK2 induced apoptosis and reduced phosphorylation of Akt, we next analyzed whether 3-phosphoinositide dependent protein kinase-1 (PDK1), which we previously showed to function as an activator of the Akt cell survival pathway in podocytes^43^, is linked to podocyte apoptosis induced by CDK2 depletion. We knocked down either CDK2 or PDK1 using lentiviral shRNAs. Knockdown of CDK2 reduced the expression of PDK1 by 48% (Fig. 8A,B), which suggests that CDK2 affects the PI3K/Akt –mediated cell survival pathway by regulating PDK1 expression. PDK1 knockdown, in turn, reduced the expression of CDK2 by 33% (Fig. 8C,D) suggesting a regulatory loop between CDK2 and PDK1 (Fig. 8E).

## Discussion

Numerous studies have reported that podocyte loss due to apoptosis or detachment is an important indicator whether podocyte injury leads to proteinuria and progressive glomerulosclerosis in diabetic and non-diabetic renal disease^5^,^45^,^46^. Therefore preventing podocyte loss in patients with glomerular disease is an important therapeutic target. In this study we found that the cell cycle regulatory protein CDK2 is expressed in the glomerular podocytes and protects podocytes from apoptosis. Previous studies reported negligible expression of CDK2 in glomeruli^47^,^48^. The difference between these studies and ours may be due to different antibodies and experimental conditions used. Furthermore, the regulation of CDK2 is more complex than regulation of its absolute expression level, as CDK2 is controlled by cyclin-dependent kinase inhibitors^30^,^48^, and for example, to regulate cell cycle, CDK2 is activated by complex formation with cyclin A or E and phosphorylation in the activation loop^49^.

We observed that CDK2 is downregulated in the glomeruli of obese Zucker rats already at an early stage before the rats develop proteinuria or glomerular apoptosis^50^,^51^,^52^. We also found that downregulation of CDK2 or inhibition of its kinase activity with roscovitine in cultured podocytes induces apoptosis further supporting a protective role for CDK2. Thus far CDK2 has been assumed to play a role in proliferative podocyte injury models, *e.g*., passive Heymann nephritis and experimental crescentic glomerulonephritis, where podocyte proliferation aggravates the decline in renal function^20^,^21^. In these models, inhibition of CDK2 limited podocyte proliferation and improved renal function^20^,^21^. It thus appears that the consequences of inhibiting or downregulating CDK2 in podocytes depend on the differentiation state of the cells. In proliferative glomerular diseases, inhibition of CDK2 is beneficial to podocytes. However, in differentiated podocytes and nonproliferative glomerular diseases, such as DN, inhibition of CDK2 in podocytes may lead to or aggravate podocyte injury. These data indicate that the function of CDK2 in podocytes needs to be tightly regulated depending on the state of differentiation.

We observed that CDK2 is downregulated in experimental podocyte injury models *in vitro*, including high glucose, PA- and LPS-induced injury. Of these, the LPS-induced podocyte injury is especially interesting in relation to DN, as we found previously that high serum LPS activity in normoalbuminuric patients with T1D predicts the progression of the disease^13^ and that the incidence of bacterial infections also correlates with the severity of DN in Finnish patients with T1D^53^. Interestingly, treatment of podocytes with sera with high LPS activity downregulated CDK2, indicating that sustained expression of CDK2 may be clinically significant in maintaining normal podocyte function. Still, we cannot rule out that some other factors in human sera in addition to LPS could contribute to downregulation of CDK2. However, high glucose did not contribute to increased apoptosis or decreased CDK2 expression in podocytes treated with human sera, since blood glucose values were similar in patients with low and high serum LPS.

The results showing that human sera with high LPS decreases CDK2 expression also provides options for treatment via blockade of the TLR pathway, as LPS binds to TLR4 that is expressed in podocytes^12^,^32^,^39^. Of note, TLR4 has been shown to be necessary for LPS responsiveness as mice in which TLR4 is mutated^40^,^54^, or mice that lack TLR4^55^, are low responders to LPS. Jialal *et al.*^56^ also demonstrated that in streptozotocin-induced diabetic mice with global TLR4 deficiency, renal inflammation, podocytopathy and fibrosis are improved^56^. Indeed, we found that in both cultured podocytes *in vitro* and mice *in vivo*, treatment with the TLR pathway inhibitor GIT27 prevented LPS-induced downregulation of CDK2.

Phosphorylation of Akt is central to the activation of the antiapoptotic PI3K/Akt cell survival pathway. CDK2 locates downstream of Akt, but is known to phosphorylate Akt on Ser477 and Thr479 promoting its activation at a specific stage during cell cycle progression^27^. We found that knockdown of CDK2 in podocytes reduced phosphorylation of Akt on Ser473 and induced apoptosis. In addition, inhibiting the kinase activity of CDK2 by roscovitine induced apoptosis in podocytes. Supporting our finding, inhibition of CDK2 in HeLa cells by roscovitine led to a significant reduction in Akt phosphorylation in different phosphorylation sites, including Ser473^27^. Furthermore, CDK2 associates with cyclin A2, and in brain-specific cyclin A2 knockout mice, Akt phosphorylation on Ser477 and Thr479 was inhibited in olfactory bulbs and led to elevated cleavage of caspase-3 and increased cellular apoptosis^27^. This further supports the idea that in the brain, CDK2/cyclin A2 complex enhances cellular survival by promoting activation of Akt. In addition, we noticed that knockdown of CDK2 led to downregulation of cyclin E, which is an activator CDK2^49^ confirming the association of cyclin E and CDK2 expression in podocytes.

PDK1 is the key mediator of the activity of the PI3K/Akt pathway downstream of PI3K and upstream of Akt and serves as a major regulator in Akt signaling^57^. We have previously shown that PDK1 protects podocytes against apoptosis^43^. Interestingly, we observed that knockdown of CDK2 in podocytes reduces the expression of PDK1 providing a novel mechanistic insight how CDK2, in addition to directly phosphorylating Akt^27^, regulates the activity of the cell survival pathway. Furthermore, we observed that knockdown of PDK1 reduces the expression of CDK2, suggesting a regulatory loop between these two proteins (Fig. 8E). Thus one can envisage that downregulation of CDK2 may further enhance the reduction of its own level via downregulation of PDK1. This apparently further aggravates the reduced activity of the PI3K/Akt pathway.

In summary, we demonstrated a novel function for CDK2 in podocytes as an antiapoptotic protein. Downregulation of CDK2 in the glomeruli of obese Zucker rats prior to significant glomerular apoptosis^43^ and development of albuminuria further suggest that downregulation of CDK2 plays a role in the diabetic kidney complication. The data also indicate a potential role for the blockade of the TLR pathway in the treatment of DN. The fact that the TLR pathway inhibitor GIT27 has been shown to be non-toxic and its administration both *i.p* and *p.o.* are equally efficient, supports its potential for clinical use^58^.

## Materials and Methods

### Animal studies

Male wild type C57BL/6JOlaHsd mice (Harlan Laboratories, Indianapolis, IN) were used to study CDK2 expression. At time of sacrifice, mice were perfused with phosphate buffered saline (PBS) for 20 minutes at room temperature (RT) before collecting the kidneys. Glomeruli were isolated by graded sieving^59^ from male Sprague-Dawley or obese (fa/fa) and lean (fa/+) Zucker rats (Crl:ZUC-Leprfa, Charles River Laboratories, Sulzfeld, Germany). Blood glucose and urinary albumin to creatinine ratio of the Zucker rats were measured as previously described^51^. Female BALB-C mice (BALB/cAnNCrl) were purchased from Scanbur (Karlslunde, Denmark). Mice were devided to three groups and treated with LPS (Sigma-Aldrich, St. Louis, MO) and GIT27 (Tocris Bioscience, Bristol, UK), LPS and PBS (vehicle for both LPS and GIT27), or PBS only (n = 6 in each group). Mice were injected intraperitoneally with GIT27 at a dose of 20 mg/kg or equal volume of vehicle. 24 h after GIT27 injection, LPS was administered at a dose of 12 mg/kg intraperitoneally. Control mice received vehicle alone. Another dose of GIT27 or vehicle was injected 2 h after LPS administration. Mice were sacrificed and kidneys harvested and processed for immunohistochemistry or electron microscopy 24 h after LPS injection. Animal procedures were approved by the National Animal Experiment Board, and all animal experiments were performed according to approved guidelines.

### Cell culture

Immortalized human podocytes^60^ were maintained as previously decribed in RPMI media supplemented with 10% fetal bovine serum (FBS), 1% glutamine, and insulin, transferrin and sodium selenite (ITS) at +33 °C for proliferation. For differentiation, podocytes were transferred to non-permissive conditions at +37 °C for 14 days. HEK293FT cells (Invitrogen, Carlsbad, CA) were maintained in DMEM medium, supplemented with 10% FBS, ultraglutamine, penicillin and streptomycin. Media, FBS and ITS were obtained from Sigma-Aldrich and ultraglutamine from Lonza (Basel, Switzerland).

### Immunoblotting

Immunoblotting was performed as decribed in^51^. Primary antibodies used were mouse monoclonal anti-CDK2 (Santa Cruz Biotechnology, Dallas, Texas, USA), rabbit polyclonal anti-CDK2 (Abcam, Cambridge, UK), rabbit polyclonal anti-phospho-CDK2 (Cell Signaling Technology, Danvers, MA), rabbit polyclonal anti-cyclin E2 (Cell Signaling Technology), rabbit polyclonal anti-CDK4 (Cell Signaling Technology), rabbit polyclonal anti-p38 MAPK, rabbit polyclonal anti-phospho-p38 MAPK (Thr180/Tyr182), rabbit polyclonal anti-phopho-Akt (Ser473), rabbit polyclonal anti-cleaved caspase-3 (Cell Signaling Technology), mouse monoclonal anti-Pan Akt (R&D Systems, Minneapolis, MN), rabbit polyclonal anti-Bax and rabbit polyclonal anti-Bcl2 (Abcam) and mouse monoclonal anti-tubulin IgGs (Sigma-Aldrich). Alexa Fluor 680 (Invitrogen) and IRDye 800 (LI-COR, Lincoln, NE) donkey anti-rabbit, anti-goat or anti-mouse IgGs were used as secondary antibodies. Detection and quantification was performed with an Odyssey Infrared Imager (LI-COR). Full Western blots with antibodies against CDK2 and phospho-CDK2 are shown in Supplementary Figure S5.

### Immunofluorescence microscopy

Differentiated human podocytes were fixed with 2% paraformaldehyde (Electron Microscopy Sciences, Hatfield, PA) in PBS and permeabilized with 0.1% Triton X-100 in PBS. Podocytes were blocked with CAS-block (Invitrogen), stained with mouse anti-CDK2 (Santa Cruz Biotechnology) for one hour at RT in ChemMate (Dako Cytomation, Glostrup, Denmark), washed with PBS and incubated with DyLight 488 donkey anti-mouse IgG (Abcam) for one hour in ChemMate (Dako Cytomation). Perfused mouse kidney samples were fixed with 10% formaldehyde and embedded in paraffin. Deparaffinized sections were blocked with CAS-block (Invitrogen), incubated with rabbit anti-CDK2 (Abcam), guinea pig anti-nephrin (Progen Biotechnik GmbH, Heidelberg, Germany) or mouse anti-WT1 (Upstate, New York, USA) IgGs overnight at +4 °C in ChemMate (Dako Cytomation), washed with PBS and incubated with AlexaFluor 555 donkey anti-rabbit (Invitrogen), DyLight 488 donkey anti-quinea pig IgGs (Jackson Immuno Research Laboratories Inc., West Grove, PA) or DyLight 488 donkey anti-mouse IgGs (Jackson Immuno Research Laboratories Inc.). Samples were mounted in Vectashield (Vector Laboratories, Burlingame, CA) and examined with Leica TCS CARS SP8 confocal microscope (Leica Microsystems CMS GmbH, Mannheim, Germany).

### Induction and detection of apoptosis

Differentiated human podocytes were exposed to puromycin aminonucleoside (PA, Sigma-Aldrich, 50 μg/ml) or lipopolysaccharide (LPS, 100 ng/ml *Escherichia coli* 0111:B4, Sigma-Aldrich) for 48 hours. The activity of LPS (100 ng/ml) in cell culture media was measured as described in^13^, and was shown to be 1.7 EU/ml. GIT27 (Tocris Bioscience) was added to the cells 2 hours before LPS exposure at a concentration of 10 μg/ml to inhibit LPS pathway receptor TLR4. Apoptosis was detected by flow cytomerty using Annexin V-FITC and 7-AAD double staining with FACSAria (BD Biosciences, Franklin Lakes, NJ). Cells positive for AnnexinV-FITC and negative for 7-AAD were deemed apoptotic. A total 1 × 10^4^ cells were detected by FACS in each sample. Apoptosis was also detected by In-Cell Western as described below.

### In-Cell Western

Podocytes cultured on black 96-well plates (PerkinElmer, Waltham, MA, USA) were fixed with 4% paraformaldehyde (Electron Microscopy Sciences) in PBS and permeabilized with 0.1% Triton X-100 in PBS. Cells were incubated with rabbit anti-cleaved caspase-3 (Cell Signaling Technology), rabbit polyclonal anti-phospho-CDK2 (Cell Signaling Technology), or rabbit anti-CDK2 (Abcam) at room temperature for one hour, followed by IRDye 800 (LI-COR) donkey anti-rabbit IgG and 1 μM DRAQ5^TM^ (Thermo Fisher Scientific, Waltham, MA, USA) at RT for one hour. Detection and quantification were performed with an Odyssey Infrared Imager (LI-COR). DRAQ5^TM^ was used for normalization.

### Electron microscopy

Cortical mouse kidney samples were fixed in 1.5% glutaraldehyde, 3% PFA and 5% sucrose in 0.1 M phosphate buffer (pH 7.4) for 3 hours at room temperature, followed by postfixation in 1% osmium tetroxide for 2 h, stained en-bloc in 1% uranyl acetate in 10% ethanol for 1 h, dehydrated in ethanol and embedded in LX-112 (Ladd Research Industries, Williston, VT). Thin sections were stained with uranyl acetate and lead citrate and examined with a JEM-1400 Transmission Electron Microscope (Jeol, Tokyo, Japan) equipped with Olympus-SIS Morada digital camera (Olympus Soft Imaging Solutions GmbH, Münster, Germany).

### Treatment of podocytes with sera obtained from patients with diabetes

Male patients with T1D were recruited and examined by the Finnish Diabetic Nephropathy Study (FinnDiane; www.finndiane.fi). Serum LPS activity levels were measured in 39 T1D patients with normal urinary albumin excretion (AER < 30 mg/24 h) as previously described^13^. Serum samples were selected from patients with highest (n = 6) and lowest (n = 6) LPS-activity (Supplementary Table S1). Differentiated human podocytes were treated with 10% human sera for 72 hours. TLR pathway inhibitor, GIT27 (Tocris Bioscience) was added to the media at a concentration of 10 μg/ml 2 hours before addition of sera from patients with normoalbuminuria and high LPS activity. The expression level of CDK2 and apoptosis were analyzed by immunoblotting and In-Cell Western, respectively, as described above. The study protocols were approved by the local ethics committees and were in accordance with the declaration of Helsinki. The study subjects gave their written informed consent to their participation in the study.

### Lentiviral infection to knock down CDK2 and PDK1

Human lentiviral pLKO1-shCDK2 vectors, CDK2A (CTCCTGGGCTGCAAATATTAT) and CDK2B (CCTCAGAATCTGCTTATTAAC) were used to knock down CDK2, and pLKO1-shPDK1 vector, PDK1A (GAAGGTATATTAGGACATTTG) (Biomedicum Genomics, University of Helsinki, Finland), to knock down PDK1 in differentiated human podocytes. Infection efficiency of the shRNA construct for PDK1 is described previously^43^. Human lentiviral empty pLKO1 vector (Biomedicum Genomics) was used as a control. For virus production, CMVDelta8.9 and phCMVg packaging plasmids (Biomedicum Genomics), together with CDK2A, CDK2B, PDK1A or empty vector, were transfected into HEK293FT cells (Invitrogen) with Lipofectamine2000 (Invitrogen). Virus-containing media were collected 72 h later, filtrated through 0.45 μm filter, and ultracentrifuged 85,000 × g at +4 °C for 90 min. The viruses were resuspended in PBS and added to differentiated podocytes on day 10 or 11 of differentiation. Podocytes were incubated with viruses at +37 °C for 10 min, followed by centrifugation 1360 × g at +4 °C for 30 min. Virus-containing medium was replaced with regular medium after 24 h.

### Roscovitine treatment

Differentiated human podocytes were cultured on black 96-well plates (PerkinElmer) at +37 °C for 24 hours in RPMI medium containing or not 25 μM roscovitine. The expression levels of phosphorylated CDK2, CDK2 and cleaved caspase-3 were detected by In-Cell Western as described above.

### Statistical methods

All cell culture experiments were carried out at least three times. All variables were presented as mean ± SD (cell culture experiments), or as mean ± SEM (animal experiments). The significance of differences between groups was determined by ANOVA or Students *t*-test (GraphPadPrism6, GraphPad Software Inc, La Jolla, CA, USA), and p-values of less than 0.05 were considered statistically significant.

## Additional Information

**How to cite this article**: Saurus, P. *et al.* Cyclin-dependent kinase 2 protects podocytes from apoptosis. *Sci. Rep.* **6**, 21664; doi: 10.1038/srep21664 (2016).

## Supplementary Material

Supplementary Information
