# Supplementary material for: Cyclin-dependent kinase 2 protects podocytes from apoptosis
Source: Sci Rep. 2016 Feb 15;6:21664. doi: 10.1038/srep21664 (PMC4753499; doi:10.1038/srep21664)
Supplement: Supplementary Information [file srep21664-s1.pdf]

Cyclin-dependent kinase 2 protects podocytes from apoptosis

Pauliina Saurus, Sara Kuusela, Vincent Dumont, Eero Lehtonen, Christopher L. Fogarty, Mariann I. Lassenius, Carol Forsblom, Markku Lehto, Moin A. Saleem, Per-Henrik Groop, and Sanna Lehtonen

Supplementary Figure S1. CDK2 is expressed in glomeruli in mouse kidney. (**A-B**) Perfused mouse kidney sections stained with an antibody against CDK2 (**A**) and with secondary antibody only (**B**). Signal for CDK2 is observed in the glomeruli (**A**) whereas no signal is seen with the secondary antibody only (**B**) confirming the specificity of the staining. Scale bars: (**A-B**) 25 µm.

Supplementary Figure S2. GIT27 has no effect on CDK2 expression in cultured human podocytes. (**A**) Representative immunoblot for CDK2 in control podocytes and podocytes treated with GIT27. Tubulin is included as a loading control. (**B**) Quantification of CDK2 in control and GIT27-treated podocytes shows that CDK2 expression level does not change after GIT27-treatment. Data are presented as mean ± SD (n=9 per treatment group), ns: non significant.

Supplementary Figure S3. CDK2 expression is downregulated in human podocytes treated with high glucose. (**A**) Quantification of In-Cell Western of CDK2 in podocytes treated with high glucose shows that the expression of CDK2 is lower after high glucose treatment. DRAQ5™ was used for normalization. Differentiated human podocytes were cultured at +37◦C for 72 hours in RPMI medium containing 11 mM (control) or 30 mM glucose (high glucose). CDK2 expression was detected by In-Cell Western using DRAQ5™ for normalization. Detection and quantification were performed with an Odyssey Infrared Imager (LI-COR, Lincoln, NE). The experiment was

performed three times. Data are presented as mean ± SD (n=24 per treatment group), ***$p < 0.001$, Student's t-test.

Supplementary Figure S4. Inhibition of the TLR pathway prevents LPS-induced downregulation of CDK2 in BALB-C mouse glomeruli. (**A-C**): CDK2 staining in control (**A**), LPS-treated (**B**) and LPS- and GIT27- treated (**C**) mouse glomeruli. CDK2 expression was lower in LPS-treated mice compared to controls and blockage of the TLR pathway prevented this downregulation. We were unable to quantify the immunohistochemistry staining of CDK2 due to non-spesific staining of red blood cells. Scale bar (**A-C**): 20 μm. In (**A-C**), mouse kidney samples were fixed with 10% formaldehyde and embedded in paraffin. Deparaffinized sections were stained with rabbit polyclonal anti-CDK2 IgG (Bethyl Laboratories, Montgomery, TX) followed by detection with VectaStain Elite ABC kit (Vector Laboratories, Burlingame, CA) and AEC (Dako Cytomation, Glostrup, Denmark). Slides were counterstained with hematoxylin and photographed using Nikon Eclipse 800 microscope (Nikon Instruments Europe BV, Amsterdam, Netherlands) using the same microscope settings throughout the analysis.

Supplementary Figure S5. Western blots with antibodies directed against CDK2 and phospho-CDK2. (**A)** Western blot of differentiated human podocyte lysates with mouse anti-CDK2 (Santa Cruz Biotechnology, Dallas, Texas, USA) and rabbit anti-CDK2 (Abcam, Cambridge, UK) IgGs. (**B)** Western blot of mouse kidney cortical lysates with rabbit anti-CDK2 IgG (Abcam). (**C**) Western blot of Zucker rat glomerular lysates with mouse anti-CDK2 IgG (Santa Cruz Biotechnology). (**D**) Western blot of differentiated human podocyte lysates with rabbit anti-phospho-CDK2 IgG (Cell Signaling Technology, Minneapolis, USA).

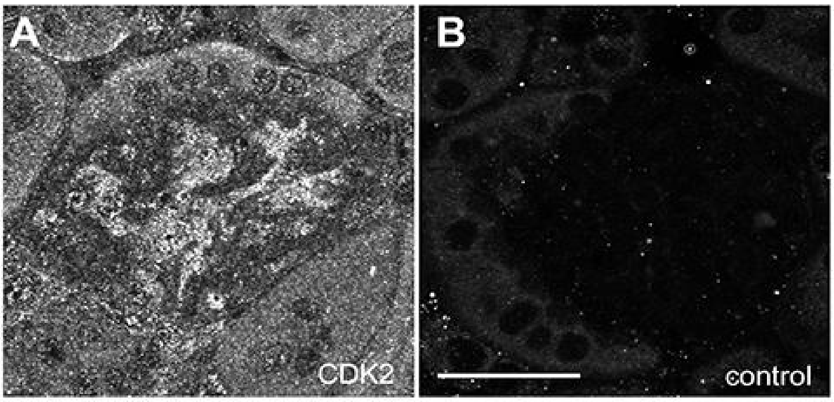

Supplementary Figure S1

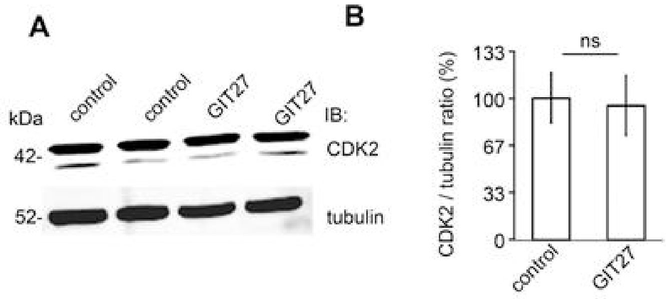

Supplementary Figure S2

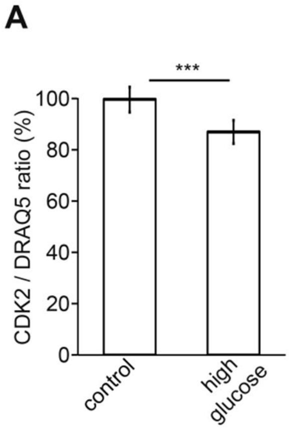

Supplementary Figure S3

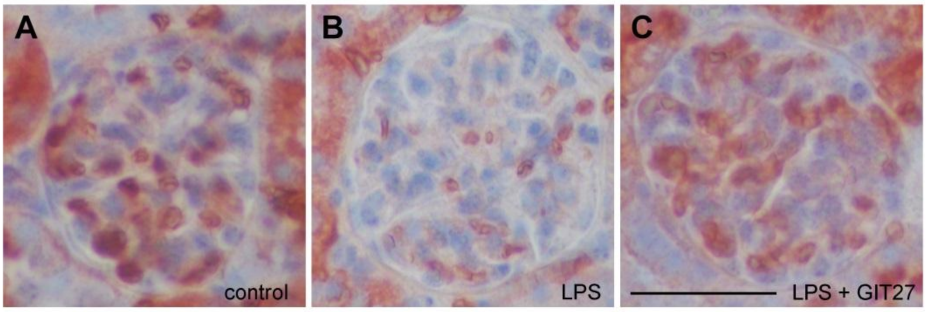

Supplementary Figure S4

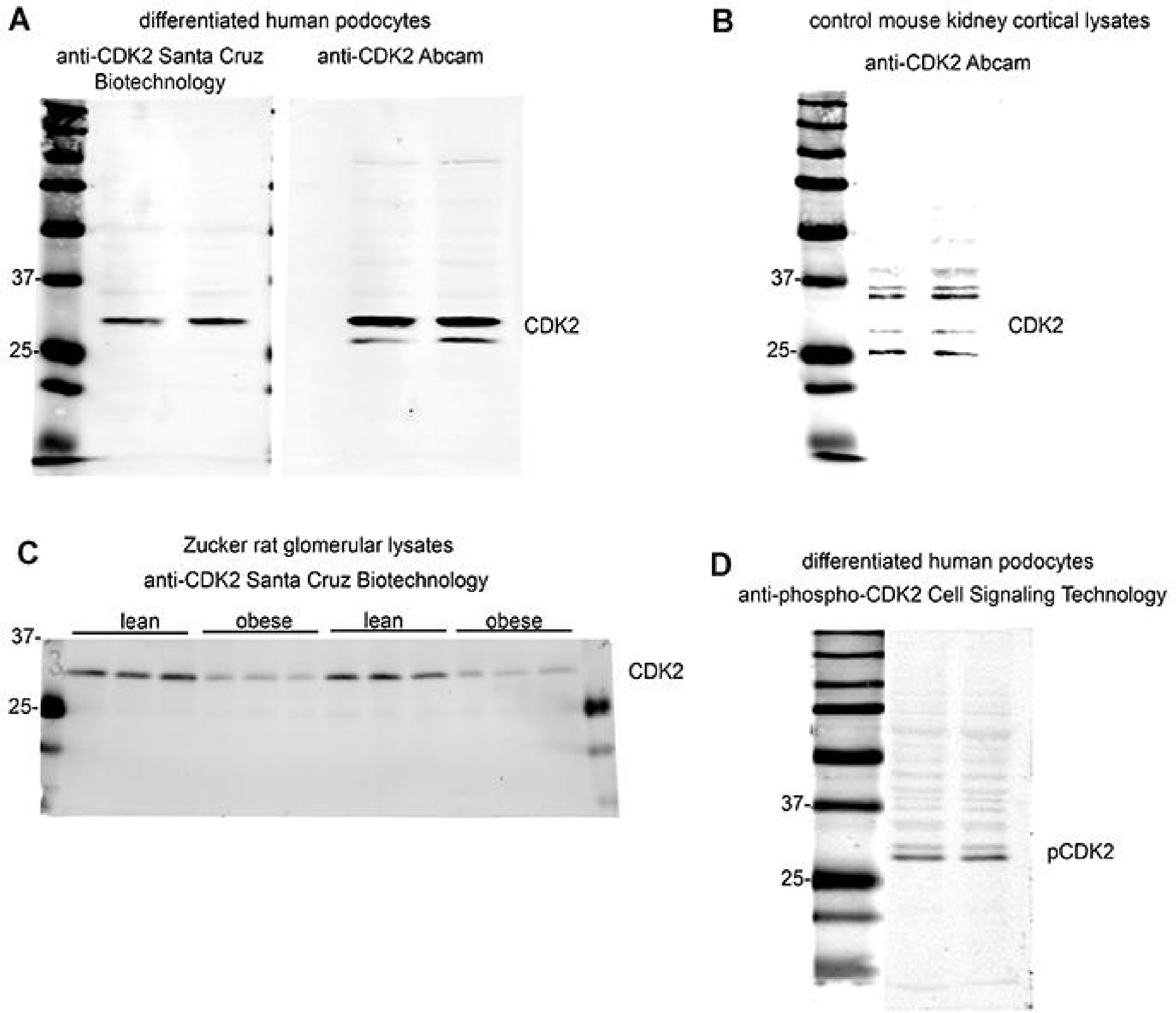

Supplementary Figure S5

Supplementary Table S1. Blood glucose and urinary albumin to creatinine values of Zucker rats.

	Blood glucose mmol/l (n=6)	Urine albumin to creatine mg/mg
Lean Zucker, 12 weeks	6.5 ± 0.7	0.013 ± 0.001 (n=5)
Obese Zucker, 12 weeks	7.5 ± 1.6	0.6 ± 0.3 (n=6) *
Lean Zucker, 40 weeks	5.2 ± 0.7	2.6 ± 2.3 (n=3)
Obese Zucker, 40 weeks	6.1 ± 1.1	25.0 ± 13.6 (n=6) *

* $p < 0.05$, Student’s t-test.

Supplementary Table S2. **Clinical characteristics of normoalbuminuric patients with T1D with low or high serum LPS activity.** Normoalbuminuria was defined as urinary albumin excretion rate <30 mg/24 h. All patients were males. LPS, lipopolysaccharide; HbA1c, hemoglobin A1c; Duration, duration of diabetes.

LPS	Low<br>(n=6)	High<br>(n=6)
Age (years)	34.8 ± 3.3	33.2 ± 6.7
Duration (years)	13.8 ± 2.7	14.2 ± 3.9
LPS (EU/ml)	0.20 ± 0.01	0.41 ± 0.04*
Triglycerides (mmol/L)	1.0 ± 0.9	1.2 ± 0.6
HbA1c (%)	7.9 ± 0.8	7.7 ± 0.2
Glucose (mmol/L)	8.63 ± 2.19	5.83 ± 1.08

* $p < 0.001$, Student’s t-test.
